# Breaking barriers: addressing transphobia and advancing transgender rights in the Asia‐Pacific and beyond

**DOI:** 10.1002/jia2.26273

**Published:** 2024-05-16

**Authors:** Rena Janamnuaysook, Danvic Rosadiño, Erika Castellanos

**Affiliations:** ^1^ Institute of HIV Research and Innovation Bangkok Thailand; ^2^ Amsterdam UMC, University of Amsterdam Department of Global Health, and Amsterdam Institute for Global Health and Development Amsterdam the Netherlands; ^3^ Amsterdam Institute for Immunology & Infectious Diseases Amsterdam the Netherlands; ^4^ Center of Excellence in Transgender Health Chulalongkorn University Bangkok Thailand; ^5^ LoveYourself, Inc. Manila Philippines; ^6^ Faculty of Management and Development Studies University of the Philippines – Open University Los Baños Philippines; ^7^ Global Action for Trans Equality Amsterdam the Netherlands

1

The International Day against Homophobia and Transphobia annually memorializes the rights violations of transgender people, yet once a year will not be enough to remember the atrocities that transgender people have to face and endure every single day. Globally, perspectives on transphobia are shaped by cultural, legal and social contexts. In multiple countries, there is a growing recognition of transgender rights, with laws evolving to protect against discrimination and hate crimes [1]. However, transphobia remains pervasive, affecting access to healthcare, education, employment and social acceptance [2].

In the Asia‐Pacific region, transgender people often face severe social stigma, legal penalties, and even violence, with little to no legal protection [3]. In some instances, colonial legacies have left enduring impacts on societal views towards gender diversity, further entrenching transphobia [4]. Transgender communities in the Philippines and Thailand have been historically struggling to battle for equality. In the Philippines, the “Equality Law” was first introduced in 2007, which is an anti‐discrimination bill based on sexual orientation, gender identity, gender expression and sex characteristics (SOGIESC). Due to the ongoing failure to pass the law, it has since been repeatedly refiled. In Thailand, the “Gender Equality Act” was enacted by the national government in 2015, which broadly promoted gender equality. In addition, Thailand recently passed a same‐sex marriage bill that the lower house of the Parliament approved by an overwhelming majority [5].

Although some Asian countries, like India, recognize transgender people as a separate gender for legal documents, many others do not, reflecting the institutionalized transphobia persisting across the region. This leaves individuals vulnerable to pervasive stigma and discrimination [6], fuelled by entrenched fears of identity falsification and deeply ingrained transphobic attitudes rooted in hetero‐cis‐normative beliefs [7]. Negative media representations further exacerbate these challenges, often linking transgender identities with stereotypical depictions of sex work and violence [8].

Notably, only 23 out of 193 United Nations member states legislated legal gender recognition based on self‐identification [9]. Without legal gender recognition in many countries in Asia and the Pacific, transgender people find it challenging to access public services and healthcare, due to transphobic environments. This can include client intake forms with binary gender options, judgemental attitudes from healthcare providers and a lack of transgender‐competent care services [10]. In the Philippines, the transgender community faces difficulties in accessing medical services in the country. Gender‐affirming hormone therapy and surgery can only be accessed by those who can afford its costs. Although hormones are widely available without a prescription in Thailand, transgender people have to self‐purchase the hormones since gender‐affirming care is not covered under the country's Universal Health Coverage scheme. Barriers to accessing this care through public health institutions push many transgender people to rely on uncertified medical providers or resort to self‐administered treatments. This desperation stems from structural inequities, provider refusal and economic obstacles, and can lead to severe health risks, including anaphylaxis, thrombosis, amputation, and even death as a result of unsupervised hormone use in an attempt to achieve gender‐affirmation [11].

In recent years, there has been a rise in transphobia as a result of the growing anti‐gender movement [12, 13, 14], which refers to a spectrum of ideologies and actions that oppose gender diversity, equality and the rights of lesbian, gay, bisexual, transgender, intersex, queer, asexual and other sexually or gender diverse (LGBTIQA+) individuals, often under the guise of protecting traditional family values or societal norms [15]. It typically manifests through political, social and legal efforts aimed at restricting the rights and recognition of transgender and non‐binary people, as well as opposing gender studies and inclusive sex education.

A recent experience reminded us that anti‐gender activists are present in all aspects of society, including in or around those bodies enshrined within the international human rights system and in mass media platforms, enabling the damaging messages of those individuals to be magnified. The World Health Organization is planning to develop global guidelines on the health of trans and gender‐diverse people. When this was publicly announced, anti‐gender activists attacked the group of experts on transgender health and rights and trans‐affiliated organizations that were invited to participate in the guideline development process. Their actions spiralled into shock waves of transphobia towards the individual experts and the trans community as a whole. Since early 2024, those involved have had to increase their personal safety and security standards, including digital security and managing additional workloads, as a result of these online and media attacks. The stress caused to the individuals involved and the burden placed on the organizations where they work were products of the efforts of the anti‐gender movement to wear down our community and hamper progress made towards the WHO Guideline Development Group meeting, ultimately delaying the publication of this much‐needed guideline for trans and gender‐diverse adults.

In a line from the documentary film “Disclosure: Trans Lives on Screen,” Jamie Clayton sums up how the transgender community positions themselves in the society: “*The more positive representation there is, the more confidence the community gains, which then puts us in more danger*.” Representation matters as this helps society recognize that this community exists, but that recognition can make some feel threatened, which can lead to retaliation—often from positions of relative ignorance about the transgender community.

Efforts to combat transphobia must adopt a multifaceted approach, addressing both legal and societal barriers to equality. Legal recognition of gender identity is paramount, requiring comprehensive legislation that explicitly safeguards transgender rights, including legal gender recognition based on self‐identification is prioritized over state‐sanctioned, often binary, transgender identities. Integrating transgender‐competent care services into healthcare settings and expanding access to gender‐affirming care, such as hormone therapy and surgery, are imperative steps towards ensuring equitable healthcare provision. Additionally, efforts to combat transphobia must address intersecting forms of discrimination, including racism, classism and ableism, recognizing the compounded barriers faced by transgender people from marginalized communities. Furthermore, education and awareness campaigns are indispensable in challenging misconceptions and reducing the stigma surrounding transgender identities. These initiatives should foster empathy, understanding and acceptance of gender diversity, cultivating inclusive environments in educational institutions, workplaces and communities. Media representation also plays a significant role in shaping public attitudes towards transgender people, emphasizing diverse experiences and narratives that humanize the community.

In conclusion, combating transphobia requires collective action and solidarity from individuals, communities and governments worldwide. By challenging discriminatory attitudes and policies, fostering inclusive environments, and advocating for legal and social recognition of transgender rights, we can aspire towards a world where all individuals are free to live authentically and without fear of discrimination or violence.

## COMPETING INTERESTS

RJ received speaker's bureau fees and research grants to her organization from Gilead Sciences. DR and EC declare no competing interests.

## AUTHORS’ CONTRIBUTIONS

RJ conceived the manuscript. DR and EC reviewed and provided inputs. All authors approved the final manuscript.
